# High prevalence of hyperlipidaemia in patients with AV re-entry tachycardia and AV nodal re-entry tachycardia

**DOI:** 10.1038/s41598-019-47940-9

**Published:** 2019-08-08

**Authors:** Ivan Zeljković, Kristijan Đula, Alen Babacanli, Ivan Kruljac, Vito Mustapić, Diana Delić Brkljačić, Nikola Bulj, Vjekoslav Radeljić, Šime Manola, Nikola Pavlović

**Affiliations:** 1 Department of Cardiology, Sestre milosrdnice University Hospital Centre, Zagreb, Croatia; 2 Department of Emergency Medicine, Sestre milosrdnice University Hospital Centre, Zagreb, Croatia; 3 Department of Internal Medicine, Sestre milosrdnice University Hospital Centre, Zagreb, Croatia; 4 School of Medicine, University of Zagreb, Zagreb, Croatia

**Keywords:** Arrhythmias, Dyslipidaemias

## Abstract

Diet rich in lipids and hyperlipidaemia increases incidence of atrial premature beats and all supraventricular arrhythmias. The aim of the study was to investigate the prevalence of hyperlipidaemia in patients with AV re-entry tachycardia (AVRT) and AV nodal re-entry tachycardia (AVNRT). We conducted a retrospective, cross-sectional, case-control study that included all consecutive patients for whom AVRT or AVNRT was confirmed during electrophysiology study. Age and gender-matched patients admitted to hospital or outpatient clinic for various reasons were randomly included and served as a control group. Hyperlipidaemia was defined according to 2016 European Society of Cardiology guidelines. A total of 1448 subjects were included: 725 patients with AVRT/AVNRT and 723 controls. AVRT/AVNRT patients had high hyperlipidaemia prevalence, which was significantly higher when compared to the control group (50.1 vs. 35.8%, p < 0.001). AVRT patients, with median age of 37.5 years, had hyperlipidaemia prevalence of 45.7%. In a multivariate analysis, hyperlipidaemia was independently associated with AVRT/AVNRT (OR 2.128, p < 0.001), both with AVNRT (OR 1.878, p < 0.001) and AVRT (OR 2.786, p < 0.001). Hypercholesterolemia was significantly more prevalent in patients with AVNRT and AVRT, while this was not the case for hypertriglyceridemia. There were no differences between the AVRT and AVNRT patients regarding hyperlipidaemia prevalence (51.9 vs. 45.7%, p = 0.801), even though AVRT patients were significantly younger (37.5 vs. 48.5, p < 0.001). In conclusion, this is the first study that investigated hyperlipidaemia prevalence in patients with AVRT or AVNRT. AVRT/AVNRT patients had higher prevalence of hyperlipidaemia and higher total and LDL cholesterol levels.

## Introduction

Nowadays, incidence of supraventricular tachycardias (SVTs) is increasing, mostly on the account of atrial fibrillation (AFib)^1,2^. The two most common paroxysmal regular SVTs are atrioventricular nodal re-entry tachycardia (AVNRT) and atrioventricular re-entry tachycardia (AVRT) using an accessory pathway (AP)^3,4^. According to the latest World Health Organization’s (WHO) report, hyperlipidaemia affects general population in epidemic measures^5^. Its prevalence is the highest in Western Europe, but is also very high across Europe as well, reaching 45–55%^5–7^. However, it is much lower in young adulthood population^8,9^. Interestingly, diet rich in lipids has been found to cause sympathetic hyperinflation and increase incidence of all supraventricular arrhythmias^10^. Moreover, several studies have shown that statins and omega-3 fatty acids regulate sympathetic activity, thereby reducing the burden of atrial premature beats, which are the most common triggers for SVT episodes^10–13^. Potential correlation between hyperlipidaemia and AVNT and AVRT has not been investigated at all. Therefore, we sought to investigate the prevalence of hyperlipidaemia in patients with APs and patients with AVNRT. We have hypothesized that symptomatic AVNRT and AVRT are associated with higher serum total and LDL cholesterol levels and higher prevalence of hyperlipidaemia.

## Methods

### Design and study population

Patients enrolled in the SVT Ablation Registry between July 2012 and June 2017, were analysed. We analysed in a retrospective, non-randomized fashion all consecutive patients that were treated for regular SVT or episodes of palpitations and were diagnosed with AVNRT, Wolff-Parkinson-White (WPW) syndrome, AVRT or solely ventricular pre-excitation. Diagnosis was confirmed during the electrophysiology study (EPS) using standard manoeuvres^14^. Patients with non-inducible tachycardia, no ventricular pre-excitation, no proof of dual AV nodal conduction physiology or accessory pathway conduction, no clear diagnosis after EPS and/or diagnosed with ventricular arrhythmias were excluded from the analysis. Baseline demographic characteristics, medical history and standard laboratory results were collected using the hospital’s patient database. Also, EPS procedural data were assessed using the SVT Ablation Registry. Analysed laboratory results during hospitalization due to EPS, included: haemoglobin, creatinine, creatine-kinase, total cholesterol, LDL cholesterol, HDL cholesterol and triglycerides. During EPS, AVNRT was diagnosed in patients with dual AV node conduction (existence of fast and slow pathway) with documented or induced clinical tachycardia: slow-fast, slow-slow and fast-slow type according to international guidelines^4,15^. AVRT was diagnosed in patients with solely ventricular pre-excitation, concealed AP with documented or induced clinical tachycardia and WPW syndrome^4,15^.

To evaluate the prevalence of hyperlipidaemia in patients with AVNRT and AVRT, we constructed a control group matched by age and sex. Patients for the control group were recruited from the hospital’s patient database, mostly from Internal medicine’s outpatient clinic which provides systematic-preventive examinations for the general population. Patients with history of palpitations or proved AVNRT or AVRT were not included in the control group. Also, due to possible influence on symptoms of palpitations and on values of serum lipids, patients with anaemia (haemoglobin <100 g/L), significant renal dysfunction (≥III grade) and/or significant proteinuria, non-corrected hyperthyreosis or hypothyreosis and with newly diagnosed or poorly controlled diabetes mellitus were excluded from the control group and the analysis.

### Hyperlipidaemia

According to 2016 European Society of Cardiology (ESC) guidelines for treating dyslipidaemias, hyperlipidaemia is defined as either hypercholesterolemia or hypertriglyceridemia, or combined^7^. In this study, hypercholesterolemia was defined as: total cholesterol >5.5 mmol/L with LDL-cholesterol >3.5 mmol/L, or total cholesterol >5.5 mmol/L including non-HDL cholesterol >4.0 mmol/L. Hypertriglyceridemia was defined as serum triglycerides >2.0 mmol/L.

### Ethics

The study was approved by *Sisters of Charity* University Hospital Ethics Committee and the Committee waived the need to obtain informed consent since this was a retrospective study. The study protocol complied with the latest revision of the Declaration of Helsinki.

### Statistical analysis

Categorical variables were presented as absolute values and percentages. Categorical variables were compared by the chi-square with Yates corrections. Continuous data were expressed as means and standard deviations or median with corresponding interquartile range. For continuous variables, comparisons were made using Student’s T-test, or Mann-Whitney U test, as appropriate. Binary logistic regression models were used to analyse the link between hyperlipidaemia and AVNRT/AVRT. Backward conditional stepwise approach was used to determine variables independently associated with the prevalence of AVNRT/AVRT and to adjust for potential confounding factors. Two-sided P values < 0.05 were considered significant. The statistical analysis was done using SPSS Version 20 (IBM SPSS Statistics, New York, USA).

### Ethical approval

The study was approved by Hospital Ethics Committee. The study protocol complied with the latest revision of the Declaration of Helsinki.

## Results

We conducted a single-centre, retrospective, cross-sectional case-control (age- and gender-matched) study. All consecutive patients with SVT hospitalised between July 2012 and June 2017 were retrospectively analysed. After assessing inclusion and exclusion criteria, a total of 1448 subjects were included in the study: 725 patients with AVNRT or AVRT (AVNRT/AVRT-group) and 723 control subjects matched by age and gender (control-group). Among AVNRT/AVRT group, 232 patients were diagnosed with AVRT and 493 with AVNRT.

Mean age of the study population was 44.3 ± 16.8 years, 56.4% were female. Baseline characteristics of study groups are given in Table 1. Patients from the control group had significantly higher prevalence of diabetes mellitus, previous myocardial infarction and were more likely to smoke. In addition, there was no difference regarding the history of beta-blocker therapy nor antiarrhythmic drug therapy (including propafenon, verapamil, sotalol, amiodarone, flecainid) between the two groups (Table 1). There were no significant differences regarding baseline demographics, as opposed to the laboratory results: AVNRT/AVRT patients had higher haemoglobin as well as total, LDL and HDL cholesterol values. Also, prevalence of hyperlipidaemia as well as hypercholesterolemia alone were significantly higher in AVNRT/AVRT patients (Table 1).

**Table 1:** Baseline characteristics of the patients with typical supraventricular tachycardia and control group.

	AVNRT/AVRT group (n = 735)	Control group (n = 723)	P value
**Demographics**			
Age (years)	45.4 ± 17.2	43.2 ± 16.4	0.11
Sex (male)	45.1 (327)	42.6 (308)	0.492
BMI (kg/m2)	26.30 ± 4.96	26.17 ± 9.20	0.749
**History**			
Hypertension	34.7 (255)	34.2 (247)	0.595
Diabetes mellitus	7.6 (56)	14.4 (104)	<**0**.**001**
Smoking	31.2 (229)	37.6 (272)	**0**.**005**
Hyperlipidaemia	18.4 (135)	18.4 (133)	1
Statin (chronic therapy)	14.7 (108)	15.4 (111)	0.769
Coronary artery disease	5.8 (42)	7.9 (57)	0.115
Myocardial infarction	2.3 (17)	6.1 (44)	<**0**.**001**
Atrial Fibrillation	8.3 (61)	1.7 (12)	<**0**.**001**
Beta-blocker therapy	15.1 (111)	17 (123)	0.354
AAD therapy	3.7 (27)	2.6 (19)	0.295
**Laboratory results**			
Haemoglobin (g/L)	142.4 ± 14.8	136.8 ± 19.3	<**0**.**001**
Creatinine (μmol/L)	79.8 ± 16.2	83.9 ± 82.8	0.189
Creatine-kinase (U/L 37 °C)	118.6 ± 96.6	117.6 ± 112.8	0.876
Total cholesterol (mmol/L)	5.23 ± 1.15	4.79 ± 1.29	<**0**.**001**
LDL-cholesterol (mmol/L)	3.25 ± 0.99	2.90 ± 1.12	<**0**.**001**
HDL-Cholesterol (mmol/L)	1.34 ± 0.34	1.21 ± 0.38	<**0**.**001**
Triglycerides (mmol/L)	1.47 ± 0.91	1.41 ± 0.92	0.196
**Prevalence**			
Hyperlipidaemia	50.1 (363)	35.8 (259)	<**0**.**001**
Hypercholesterolemia	41.6 (302)	28.9 (209)	<**0**.**001**
Hypertriglyceridemia	22.1 (160)	18.1 (131)	0.25

Values are % (n) for categorical and mean ± standard deviation or median (25^th^–75^th^ percentile) for continuous variables. BMI- body mass index. AAD therapy - antiarrhythmic therapy (including propafenon, verapamil, sotalol, amiodarone, flecainid).

In multivariate analysis, AVNRT/AVRT was independently associated with advanced age (OR 1.024, 95% CI 1.025–1.033, p < 0.001), higher BMI (OR 1.068, 95% CI 1.035–1.103, p < 0.001), higher haemoglobin values (OR 1.03, 95% CI 1.02–1.04, p < 0.001), higher HDL cholesterol (OR 5.09, 95% CI 3.23–8.01, p < 0.001) and higher LDL cholesterol (OR 1.23, 95% CI 1.07–1.42, p < 0.001).

### AVRT and AVNRT

When comparing patients with AVRT and AVNRT, patients with AVNRT were significantly older, more likely to be women, have higher prevalence of hypertension and diabetes mellitus (Table 2). However, there was no difference in prevalence of hyperlipidaemia between these two groups (p = 0.8) (Table 2). Also, when we compared patients with AVNRT and AVRT separately with their control groups, both patients with AVRT (45.7 vs 33.7%, p < 0.001) and with AVNRT (51.9 vs 39.4%, p < 0.001) had higher prevalence of hyperlipidaemia. Hypercholesterolemia alone was also more often found in AVRT (38.4 vs 26.9%, p < 0.001) and AVNRT patients (43 vs 31.7%, p < 0.001), while this was not the case for hypertriglyceridemia (AVRT: 18.1 vs 16.7%, p = 0.28; AVNRT: 23.7 vs 19.5%, p = 0.082).

**Table 2:** Baseline characteristics and prevalence of hyperlipidaemia of AVRT patients and AVNRT patients.

	AVNRT-group (n = 493)	AVRT group (n = 232)	P value
**Demographics**			
Age (years)	48.5 ± 16.2	37.5 ± 15.6	<**0**.**001**
Sex (male)	37.3 (184)	59.9 (139)	<**0**.**001**
BMI (kg/m^2^)	26.56 ± 5.08	25.7 ± 4.68	**0**.**032**
**History**			
Hypertension	38.9 (192)	23.7 (55)	<**0**.**001**
Diabetes mellitus	9.53 (47)	1.6 (7)	**0**.**001**
Smoking	30.4 (150)	33.6 (78)	0.283
Hyperlipidaemia	17 (84)	21.1 (49)	0.217
Statin (chronic therapy)	15.2 (75)	13.4 (31)	0.632
Coronary artery disease	6.1 (30)	5.2 (12)	0.623
Myocardial infarction	2.4 (12)	2.2 (5)	1
Atrial Fibrillation	7.3 (36)	9.9 (23)	0.204
**Laboratory results**			
Haemoglobin (g/L)	141 (132, 151)	147 (138, 157)	0.067
Creatinine (μmol/L)	76 (66,87)	80 (71,94)	0.11
Creatine-kinase (U/L 37 °C)	95 (70,132)	100 (75,136)	0.109
Total cholesterol (mmol/L)	5.27 ± 1.16	5.15 ± 1.17	0.226
LDL-cholesterol (mmol/L)	3.28 ± 0.99	3.2 ± 0.99	0.325
HDL-Cholesterol (mmol/L)	1.35 ± 0.35	1.33 ± 0.32	0.569
Triglycerides (mmol/L)	1.47 ± 0.87	1.48 ± 0.98	0.591
**Prevalence**			
Hyperlipidaemia	51.9 (256)	45.7 (106)	0.801
Hypercholesterolemia	43 (212)	38.4 (89)	1
Hypertriglyceridemia	23.7 (117)	18.1 (42)	0.322

Values are % (n) for categorical and mean ± standard deviation or median (25^th^–75^th^ percentile) for continuous variables. BMI- body mass index.

### Association of hyperlipidaemia with AVNRT/AVRT and other parameters

**Figure 1:**
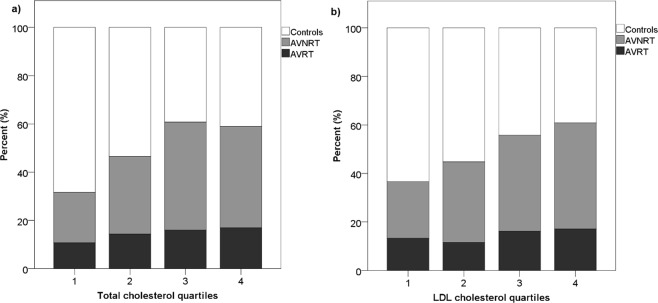
Rate of combined AVRT/AVNRT prevalence (black bars) in different quartiles of total cholesterol (**a**), and LDL cholesterol (**b**).

**Figure 2:**
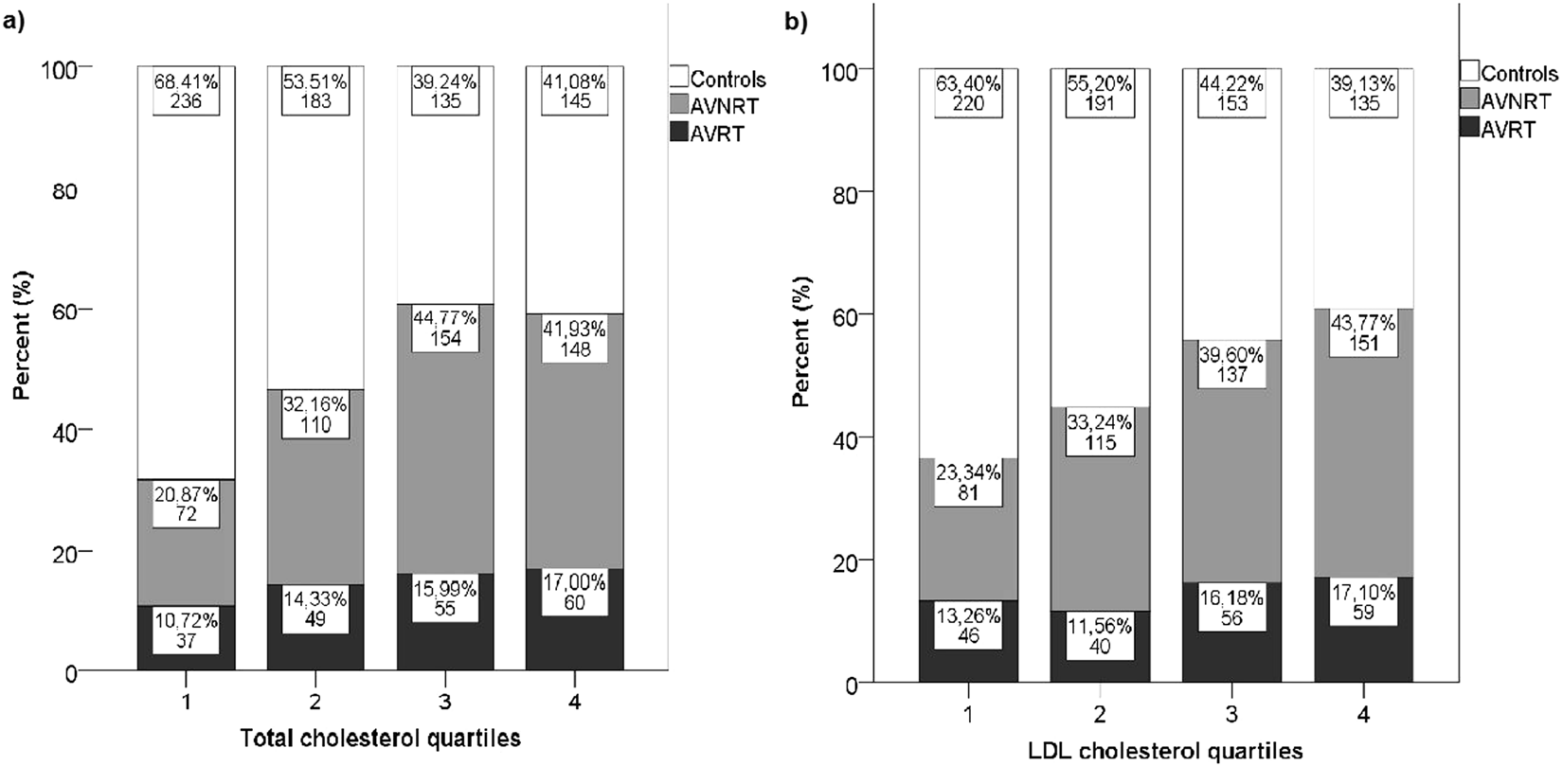
Rate of AVRT (black bars) and AVNRT (grey bars) independently in different quartiles of total cholesterol (**a**), and LDL cholesterol (**b**).

Hyperlipidaemia was present in 44.7% of all included subjects (AVNRT/AVRT patients and controls). In multivariate analysis, hyperlipidaemia was independently associated with 2-fold increase in chance for AVNRT and AVRT altogether (OR 2.128, 95% CI 1.683–2.689, p < 0.001). Similar increase was observed both for AVNRT (OR 1.878, 95% CI 1.452–2.430, p < 0.001) and AVRT (OR 2.786, 95% CI 1.965–3.949, p < 0.001). Hyperlipidaemia was also independently associated with higher body mass index (BMI) (OR 1.130, 95% CI 1.101–1.159, p < 0.001), advanced age (OR 1.018, 95% CI 1.009–1.027, p < 0.001), hypertension (OR 1.429, 95% CI 1.059–1.927, p = 0.020) and smoking (OR 1.460, 95% CI 1.145–1.863, p = 0.002), but not with coronary artery disease and previous myocardial infarction. Hypercholesterolemia alone was associated with both AVRT and AVNRT, unlike hypertriglyceridemia (Table 3). When we divided the entire study population into quartiles (Q1–4) according to values of total and LDL cholesterol, patients in Q3 and Q4 had a significantly higher prevalence of AVNRT/AVRT (Fig. 1), as well as significantly higher prevalence of AVRT and AVNRT independently (Fig. 2a,b).

**Table 3:** The final step of stepwise conditional backward binary regression showing parameters that are independently associated with hyperlipidaemia, hypercholesterolemia and hypertriglyceridemia.

	B	S.E.	P value	OR	95% CI	
**Hyperlipidaemia**						
Age	0.020	0.004	<0.001	1.020	1.011	1.029
BMI	0.122	0.013	<0.001	1.130	1.101	1.160
Hypertension	0.360	0.153	0.019	1.433	1.062	1.935
Smoking	0.371	0.125	0.003	1.449	1.135	1.851
AVNRT	0.630	0.131	<0.001	1.878	1.452	2.430
AVRT	1.013	0.178	<0.001	2.754	1.942	3.905
**Hypercholesterolemia**						
Age	0.025	0.004	<0.001	1.025	1.017	1.033
BMI	0.060	0.012	<0.001	1.062	1.037	1.087
AVNRT	0.525	0.128	<0.001	1.690	1.314	2.174
AVRT	0.881	0.173	<0.001	2.413	1.719	3.389
**Hypertriglyceridemia**						
BMI	0.143	0.015	<0.001	1.153	1.121	1.187
Hypertension	0.335	0.153	0.028	1.398	1.036	1.886
Smoking	0.415	0.147	0.005	1.514	1.134	2.020
AVNRT	0.246	0.147	0.093	1.279	0.960	1.705
AVRT	0.231	0.139	0.087	1.897	0.881	2.916
Atrial fibrillation	0.834	0.276	0.003	2.302	1.339	3.957

B - unstandardized correlation coefficient; S.E - standardised error; OR - odds ratio; CI - confidence interval;

BMI - body mass index, AVNRT - atrioventricular nodal re-entry tachycardia; AVRT - atrioventricular re-entry tachycardia.

## Discussion

To the best of our knowledge, this was the first study that investigated prevalence of hyperlipidaemia in patients with AVNRT and AVRT. The main findings of this study are the following: (1) AVNRT/AVRT patients had high prevalence of hyperlipidaemia (50.1%), which was significantly higher when compared to the control group matched by age and gender; (2) AVRT patients, with a median age of 37.5 years, had prevalence of hyperlipidaemia of 45.7%; (3) in a multivariate analysis, hyperlipidaemia was independently associated with AVNRT/AVRT altogether (OR 2.128), both with AVNRT (OR 1.878) and AVRT (OR 2.786) separately; 4) hypercholesterolemia was significantly more prevalent in patients with AVNRT and AVRT, while this was not the case for hypertriglyceridemia.

We conducted a retrospective, case-control analysis which included more than 1400 patients and the control group was matched by age and gender. However, control patients had more comorbidities including higher prevalence of diabetes mellitus, smoking and previous myocardial infarction, as well as lower levels of haemoglobin. The fact that patients with AVNRT or AVRT had significantly higher prevalence of hyperlipidaemia, despite lower prevalence of diabetes mellitus which proved to be a significant risk factor for hyperlipidaemia^7,16^, makes the results of this study even more pronounced. On the contrary, AVNRT/AVRT group had higher prevalence of AFib, which corresponds to earlier studies, and especially is typical for AVRT patients^4,17,18^. Also, our data suggest that hyperlipidemia may not be associated with myocardial infarction and coronary artery disease which could be explained with AVRT/AVNRT patients being generally younger than the general population of patients with myocardial infarction and coronary artery disease as well as the fact that the myocardial infarction and coronary artery disease prevalence in our study population was low and thus statistically underpowered to show any difference.

Patients with low haemoglobin (<100 g/L) were excluded from the analysis, and AVNRT/AVRT patients had higher median haemoglobin value compared to controls, but both groups had values significantly above the lower reference limit and it should not had any clinical impact or significance.

Prevalence of hyperlipidaemia is very high in Europe, especially in developed Western European countries, estimating to 55%, probably due to lifestyle and ageing of population^5,6^. In this study, the prevalence of hyperlipidaemia was 44.7% in a population with mean age 44.3 (±16.8) years which is in line with yearly reports. This is expected since Croatia is not as developed as Western European countries, with younger general population and possibly a large impact of Mediterranean diet^5,19^. Also, AVNRT/AVRT patients had prevalence of hyperlipidaemia of 50.1% which is high for their mean age, and significantly higher when compared to controls who had more comorbidities. Additionally, AVRT patients had prevalence of 45.7%, which is very high for their median age of 37.5 years^5^. Patients with hyperlipidaemia have higher burden of atrial premature beats which are the most common cause of AVNRT and AVRT initiation^10,11,17,20^. It is possible that patients with pre-existing dual AV nodal conduction and/or AP, more often develop tachycardia and palpitations due to hyperlipidaemia, and consequently come to hospital for EPS and are diagnosed with both entities. This could be linked to the fact that Khori *et al*. proved on an “*in vitro* model” that simvastatin had a similar effect on slow pathway conduction as verapamil, consequently terminating AVNRT^21^. However, there is a significant increase in hyperlipidaemia prevalence in general population in the last decades, but the increase in AVRT and/or AVNRT prevalence or incidence was not noticed^4,6,22^. Also, it is important to note that an increased prevalence of hyperlipidaemia was established mostly on the basis of hypercholesterolemia, while hypertriglyceridemia was not associated with AVNRT or AVRT. Ultimately, the temporal nature of this AVNRT/AVRT and hyperlipidaemia correlation remains unclear.

### Limitations

The results of the present study should be interpreted in the light of certain limitations. Firstly, this was a retrospective analysis of the single-centre data. However, more than 1400 patients overall were included, and both AVRT and AVNRT patients had age and gender matched controls, but multicenter and prospective studies are needed to confirm these results. Secondly, the prevalence of hyperlipidaemia proves to be influenced by age, sex, BMI and lifestyle. Lifestyle analysis was not conducted, but there were no differences between study groups regarding demographic characteristics. Thirdly, nowadays the management of AVNRT/AVRT is not the focus of attention; however, this correlation could help reveal if patients with AVNRT/AVRT should be screened for hyperlipidaemia, especially those of young age. Ultimately, the results refer to a relatively specific group of patients who are rather young and have low comorbidities’ incidence, and consequently we don’t know whether our findings can be found in other populations.

In conclusion, patients with AVNRT and AVRT have high prevalence of hyperlipidaemia, which is significantly higher when compared to controls matched by age and gender. Patients with Q3–Q4 values of total and LDL cholesterol have higher prevalence of AVNRT/AVRT when compared to Q1–Q2 patients. This was the first study that investigated prevalence of hyperlipidaemia in patients with AVNRT/AVRT. Since this was a retrospective, single-centre study, a further prospective, multicentre clinical studies, as well as basic research studies, are needed to confirm these results.

## Data Availability

The summarized data used to support the findings of this study have been deposited in the hospital’s internal database and registry. Also, the summarized data used to support the findings of this study are included within the article. The individual data used to support the findings of this study are restricted by the Croatian laws in order to protect patients’ privacy. Data are available from Ivan Zeljkovic, corresponding author, for researchers who meet the criteria for access to confidential data.
